# Avian models for brain mechanisms underlying altered social behavior in autism

**DOI:** 10.3389/fphys.2022.1032046

**Published:** 2022-10-28

**Authors:** András Csillag, Ágota Ádám, Gergely Zachar

**Affiliations:** Department of Anatomy, Histology, and Embryology, Faculty of Medicine, Semmelweis University, Budapest, Hungary

**Keywords:** social behavior, songbird, ASD, dopamin, valproic acid, genes

## Abstract

The current review is an update on experimental approaches in which birds serve as model species for the investigation of typical failure symptoms associated with autism spectrum disorder (ASD). The discussion is focused on deficiencies of social behavior, from social interactions of domestic chicks, based on visual and auditory cues, to vocal communication in songbirds. Two groups of pathogenetic/risk factors are discussed: 1) non-genetic (environmental/epigenetic) factors, exemplified by embryonic exposure to valproic acid (VPA), and 2) genetic factors, represented by a list of candidate genes and signaling pathways of diagnostic or predictive value in ASD patients. Given the similarities of birds as experimental models to humans (visual orientation, vocal learning, social cohesions), avian models usefully contribute toward the elucidation of the neural systems and developmental factors underlying ASD, improving the applicability of preclinical results obtained on laboratory rodents. Furthermore, they may predict potential susceptibility factors worthy of investigation (both by animal studies and by monitoring human babies at risk), with potential therapeutic consequence.

## Introduction

With many problems in biomedicine, the key to success is finding a suitable model system/species. Experimental models are essential for evidence-based studies, especially those of the interventional type (which would be impractical or ethically unacceptable to carry out in humans). They also help finding the appropriate level of explanation, blissfully avoiding the traps of Scylla and Charybdis, extreme reductionism (*cf.* the problem described as “the janitor’s dream” by Calvin, 1998) or undue generalization. Model systems, however, may fail at times. Ever since, there has been an increasing demand for alternative model species (Bolker, 2012; Yartsev, 2017) and phylogenetic comparisons to improve the poor applicability of preclinical results obtained mostly on laboratory rodents (Perrin, 2014).

Autism Spectrum Disorder (ASD) is one of the most common neurodevelopmental disorders associated with altered social behavior. The variable and multifaceted character of the disease necessitates reliable animal models and multilateral approaches. Birds as evolutionary alternatives to mammals, with a wide behavioral repertoire, may well represent a special window of observation. Animal models may help discern genetic vs*.* non-genetic (epigenetic, environmental) factors in the etiology of ASD (Ergaz et al., 2016). Experimental intervention may help reveal the pathogenetic causes during gestation, and whether such risk factors can be antagonized or reversed.

The current summary is not intended to give a comprehensive account of available animal models for different autism related syndromes (*cf.* previous thorough reviews, Ergaz et al., 2016; Nicolini and Fahnestock, 2018). Here, we are focusing on those applied on avian species, elaborating on the valproic acid model (an example for non-genetic interventions), and on a set of genetic modifications, in which birds have been used as experimental subjects. Avian models have helped in the past to solve problems such as neural plasticity in early learning (McCabe and Horn, 1994; Rose, 2000), adult neurogenesis (Goldman and Nottebohm, 1983; Nottebohm, 2005), thanks to the existence of common patterns in evolution. In many ways, birds are better models of human behavior than mammals. They are highly visual beings, display vocal communication, even vocal learning, and often live in pairs or flocks, exposed to multiple social signals.

Newly hatched domestic chicks have often been used as models in studies of behavioral neuroscience (Bolhuis and Honey, 1998; Rose, 2000; Zachar et al., 2008), because they can display complex behaviors, not confounded by earlier experience (Rose, 2000). Just like newborn humans, chicks have a predisposition to prefer the proximity of conspecifics (for review see Di Giorgio et al., 2017). Whether such predispositions are affected by autism is a matter of debate (Elsabbagh et al., 2013; Jones and Klin, 2013; Sgad**ò** et al., 2018; Zachar et al., 2019), however, the social bonds based on innate stimulus preferences are certainly impaired by ASD. The social interactions of domestic chicks are based on visual and auditory cues (Koshiba et al., 2013), i.e., traits that are more human-like than the olfactory-biased sociability of most mammals (Brennan and Kendrick, 2006).

There are striking behavioral similarities between children with ASD and domestic chicks with socio-sensory deprivation (Koshiba et al., 2016), further supporting feasibility of the avian model.

**FIGURE 1:**
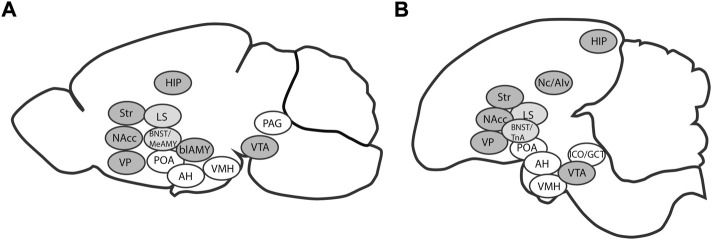
Brain regions constituting the social brain network (white), the mesolimbic dopaminergic reward system (dark grey) and their overlap (light grey) in mammals **(A)** and in birds **(B)**. The two interconnected networks are collectively designated as the social decision-making network. AH: anterior hypothalamus, blAMY: basolateral amygdala (avian homologue Nc/AIv: caudal nidopallium/ventral intermediate arcopallium), BNST: bed nucleus of the stria terminalis, HIP: hippocampus, LS: lateral septum, MeAMY: medial extended amygdala (avian homologue TnA: nucleus taeniae), NAcc: nucleus accumbens, PAG: periaqueductal gray (avian homologue ICO/GCT: intercollicular nucleus/midbrain central gray), POA: preoptic area, Str: striatum, VMH: ventromedial hypothalamus, VP: ventral pallidum, VTA: ventral tegmental area. Based on O'Connell and Hofmann, 2011.

Chicks react to social isolation by displaying behaviors aimed at reuniting with conspecifics (Gallup and Suarez, 1980), and they prefer larger groups of siblings over smaller ones (Zachar et al., 2017). The drive to reinstatement can be evaluated by measurement of distress vocalization (Marx et al., 2001; Takeuchi et al., 1996; Yazaki et al., 1999; Montevecchi et al., 1973; Zsedényi et al., 2014). Such innate gregariousness of naïve domestic chicks likely relies on the social brain network (SBN, Goodson, 2005), and affiliation to siblings is likely processed similarly to other social behaviors (Mayer et al., 2017). The mesolimbic dopaminergic reward system is amply interconnected and overlapping with SBN forming the phylogenetically conservative social decision-making network (O'Connell and Hofmann, 2011) (Figure 1). Therefore, the separation-reinstatement paradigm of the young domestic chick can be an appropriate laboratory model of sociability [e.g., the test for group preference (Zachar et al., 2017) and other tests of belongingness or aggregation, see Nishigori et al., 2013].

Song learning and singing in oscine birds is often paralleled with the human language (affected by ASD). Remarkably, however, no studies known to us have been reported on effect of embryonic VPA treatment on the vocal behavior of songbirds (either learned singing or innate calls).

Relevant VPA based (or other environmental intervention-based) models should also consider the importance of innate calls. Parental care requires intense cooperation through coordination and synchronization of behavior, and an intense communication between the parents. Zebra finches change their vocal communication over pair formation and during nest building and incubation (Gill et al., 2015). They reduce the amount of distance calls and courtship singing, while increasing the frequency of the low amplitude calls specifically used for short distance communication between the pairs (D’Amelio et al., 2017). Furthermore, the acoustic interactions between the two members of the pair become more synchronized (Gill et al., 2015). Coordinated duetting between the parents may function as negotiations over the parental effort, at least during the egg incubation phase (Boucaud et al., 2016, 2017). Compared to widely studied courtship songs, little is known about the short distance calls (Ter Maat et al., 2014). The neural substrate responsible for these calls overlaps, at least partially, with the brain’s song system (Gobes et al., 2009; Giret et al., 2015), however the different social function implies the involvement of different regions and/or genes. The elaborate male song is a learned behavior, and, in this sense, it is more like human speech than are innate calls. The shorter calls are suitable for individual recognition (Elie and Theunissen, 2018) and for promoting cooperation between individuals, another facet of similarity with human language. The latter is often neglected in the scientific literature on birdsong. Moreover, female zebra finches are also capable of cooperative vocalization. They possess a less developed (but otherwise homologous) neural network for processing song than that of males (Shaughnessy et al., 2019). Such, more specific, songbird models might provide novel insight into cooperative vocalization in normal or pathological states.

### Embryonic treatment with VPA

A well-established ASD model for laboratory rodents is prenatal exposure to valproic acid (VPA), a known antiepileptic substance (Rodier et al., 1996, for comprehensive reviews see Roullet et al., 2013; Nicolini and Fahnestock, 2018) and mood stabilizing agent (Cipriani et al., 2013). The key factor in the action of VPA seems to be inhibition of histone deacetylase (Göttlicher et al., 2001), affecting gene expression and transcription during CNS development.

VPA has been used in birds first as a teratogenic agent. Domestic chicken eggs were injected with VPA at critical times of development to study malformations (Barnes et al., 1996; Whitsel et al., 2002; Hsieh et al., 2013), or modifications of gene expression (Akhtar et al., 2015), e.g., reduction of PAX6 (Zosen et al., 2022). The reported distinct and dose-dependent effects of VPA on brain development potentially reflect on behavioral measures (Barnes et al., 1996; Zosen et al., 2022).

In relation to autism-related social deficits, administration of VPA *in ovo* caused impairment of social behavior (but not imprinting) in chicks (Nishigori et al., 2013). VPA alters the approach response to visual cues resembling to conspecifics such as simulated biological motion (Lorenzi et al., 2019; Matsushima et al., 2022) or face like stimuli (Adiletta et al., 2021), suggesting an early effect on social stimulus processing similar to autistic children. VPA abolished the innate visual predispositions of chicks to hen features, while imprinting remained unaffected (Sgad**ò** et al., 2018). Similarly, in a study by Zachar et al. (2019), early learning (passive avoidance training) and color discrimination were not impaired by VPA treatment. At variance with the results of Sgad**ò** et al. (2018), albeit at a later phase of post-embryonic development, VPA exposure did not affect the innate approach preference of birds for the larger over smaller group of conspecifics, or for companion birds with natural facial features over those with blurred features (Zachar et al., 2019). However, VPA did attenuate social exploration and the recognition of familiar conspecifics, by the end of the third week post-hatch, drawing attention to the importance of early social exploration in human ASD (Zachar et al., 2019). The corollary from these studies is that subtle alterations in innate predispositions and social exploration might well predict the future manifestation of ASD. Therefore, a standardized recording and monitoring of human babies at risk during the early postnatal period would be highly recommended practice.

The valproate model exemplifies the potential role of epigenetic/environmental factors in the pathogenesis of ASD. Other animal models, including those applied mainly in songbirds, are based upon genomic alterations.

### Genomic alterations

Deficits in the acquisition of culturally transmitted social skills, including speech and language are important early indicators of ASD (Tager-Flusberg et al., 2005; Mody and Belliveau, 2013; Sperdin and Schaer, 2016). The elaboration of birdsong is often compared to the complexity of human speech (Aamodt et al., 2019). Songbirds may represent useful models for certain aspects of ASD both in terms of vocal communication and sociability. Several candidate genes, common to songbirds and humans, have been described to participate in the production and socially meaningful perception of song/speech (for an overview of zebra finch studies see Panaitof, 2012). Genomic interventions in altricial songbirds may help understand the etiology of some of the failure symptoms in ASD. By contrast, very few studies have tackled the genetic basis of social behavior of precocial birds. Of five candidate genes, TTRAP showed a correlation with social behavior of domestic chicks (Johnsson et al., 2018). Though none of those five genes were confirmed candidates in human autism (Satterstrom et al., 2020), TTRAP is associated with language-related regions (Pinel et al., 2012).

The following account is not intended to cover the ever-expanding plethora of genomic factors that are potentially linked to the pathogenesis of ASD. We merely attempted here to summarize the most promising lines of investigation, in which songbirds played an important part as experimental subjects.

#### FOXP1, FOXP2

The Forkhead Box transcription factors FOXP1 and FOXP2 were found to be linked to speech and language disorders (Lai et al., 2001), and are among the risk genes for autism (Satterstrom et al., 2020). Similarly distributed in the developing human and songbird language-related centers, they proved to be promising candidates for cross-species studies (Teramitsu et al., 2004). Knockdown of *FoxP2* in the basal ganglia song nucleus, Area X, was found to impair singing in zebra finches (Haesler et al., 2007). The importance of *FoxP2* in the regulation of singing has been supported by other suppression or overexpression studies (Murugan et al., 2013; Heston and White, 2015). In a more recent zebra finch study, *FoxP1* was found to be expressed mainly in striatal-projecting HVC neurons (forebrain mirror neurons). Knockdown of *FoxP1* expression in juvenile birds led to a selective learning deficit, affecting the ability to form memories essential for the cultural transmission of behavior (adult model song) (Garcia-Oscos et al., 2021).

#### Cntnap2

An important target of FOXP2, Contactin-associated protein-like 2 (*Cntnap2*) (Spiteri et al., 2007) has been identified as an autism susceptibility gene (Alarcón et al., 2008). This gene is considered a risk factor for language-related disorders, including ASD, language impairment, and stuttering (Arking et al., 2008; Li et al., 2010). A specific enrichment of the CNTNAP2 protein was found in the song nuclei of male zebra finches (Condro and White, 2014), pointing to a generalized role in vocal learning across vertebrate species.

#### FXS, FMRP

Fragile X syndrome (FXS) is the most common inherited form of ASD, characterized by hyperactivity, impulsivity, and anxiety, as well as by defective language development. Many FXS symptoms appear early in life, together with emerging autistic features (Hagerman et al., 2017). A trinucleotide repeat disorder, silencing of the gene leads to the loss of its product, Fragile X mental retardation 1 protein (FMRP). FMRP is an RNA-binding protein regulating the translation of numerous mRNAs instrumental in the development and maintenance of synapses. FXS animal models are based on the loss of neural plasticity and an imbalance between inhibitory and excitatory neuronal circuits, also mimicking certain clinical symptoms of ASD. FMRP is a promising target for therapeutic intervention. The gene and its product have been identified in the vocal control system of the zebra finch, recommended as a model for FXS-associated language disorders (Winograd et al., 2008). Curiously, however, despite obvious therapeutic advantages, experimental interventions on FXS or FMRP, in relation to ASD, have not yet been reported in avian species.

In addition to songbirds, FMRP has been located also in the brainstem auditory nuclei of domestic chicks, with a specific role in dendritic dynamics (Wang et al., 2014) and axonal growth (Wang et al., 2020). In this capacity, FMRP is just one of many genomic factors to regulate axonal pathfinding, some of which have been demonstrated in avian vocal learning-relevant regions, e.g., the SLIT-ROBO system (Wang et al., 2015).

#### ADNP

ADNP is an essential protein instrumental in brain development and neural plasticity, thereby determining a host of social and cognitive functions potentially malfunctioning in autism. Mutations in ADNP system have been found in human ASD cases (Helsmoortel et al., 2014; Satterstrom et al., 2020). An established experimental model, *Adnp*± mice develop impairments of cognitive and social behaviors (Vulih-Shultzman et al., 2007), resulting in Alzheimer’s disease related symptoms, as well as autistic features (Malishkevich et al., 2015). Notably, male *Adnp*± mice are more seriously affected, mimicking a similar prevalence of failure symptoms in human subjects with ASD. The sex- and age-related expression of ADNP mRNA was reported in different areas (cerebellum, cerebrum, brainstem) of the zebra finch (Hacohen-Kleiman et al., 2015), with a distinct sexual dimorphism (young males expressing higher levels of ADNP than females, in agreement with the notion that only males perform courtship singing). The gene expression profile was largely confirmed in the domesticated canary, and ADNP mRNA was found to be enriched mainly in the mesopallium, harboring centers for sensory integration and higher auditory processing (Hacohen-Kleiman et al., 2020).

#### mTOR

Owing to its role in experience-dependent synaptic plasticity (Garza-Lombó and Gonsebatt, 2016), the Mechanistic Target of Rapamycin (mTOR) signaling cascade has been implicated as a factor in the etiology of ASD, based chiefly on mouse models (Chen et al., 2014; Kazdoba et al., 2016). In an elegant study on zebra finch (Ahmadiantehrani and London, 2017) mTOR signaling was activated in the auditory forebrain by memorization of tutor song in adult males but not in younger males (not old enough to copy song) or in females (who cannot sing). Both the inhibition and constitutive activation of mTOR during tutor experiences diminished copying of tutor song. Remarkably, constitutive mTOR activation lowered the ‘social engagement’ of juvenile zebra finches during tutor experiences, somewhat similarly to the situation found in humans with autism. The findings bear relevance for the role of the onset of mTOR cascade in the encoding of early life experience to determine future behavior.

#### Glycogen synthase kinase-3 (GSK-3)

GSK-3 is a highly conserved serine/threonine protein kinase that plays a central role in a wide variety of cellular processes associated with cognition and behavior (Beurel et al., 2015). In a recent study on zebra finch, inhibition of the splice variant GSK-3β was found to attenuate social recognition and decision making (Moaraf et al., 2022). Interestingly, birds are “natural knockouts” for the *GSK-3α* gene (Alon et al., 2011), enabling selective investigation of the effects of *GSK-3*β. Although *GSK-3*β is not among the 102 key risk genes recently defined for autism, being the 150th among 18,000 observed genes (Satterstrom et al., 2020), the findings related to social recognition in songbirds may potentially indicate a future involvement and predict a novel line of investigation in this direction.

#### Novel aspects of convergent genomic regions

Based on a meta-analysis of convergence between avian and human accelerated genomic regions (AR), the important study by Cahill et al. (2021) casts light on the regulation of vocal learning in different clades of birds (“rediscovered” two to three times during avian evolution) and that of human speech. In addition to known AR such as FOXP2 (already discussed above), further novel candidate genes were ‘mined’ in this study. For example, NR2F1, a neurodevelopment regulating transcription factor with predicted function in vocalization behavior, proved to be the highest density AR hotspot specific to vocal learning birds, and it is also a SFARI class S gene for ASD (Abrahams et al., 2013).

In most of the cases described above the genomic risk factors had been identified first in humans, then confirmed in mammalian model systems, and avian experiments “followed suit” as logical sequels. Notably, however, in the last two paragraphs examples were given for a reverse order of events: avian studies taking the lead to predict potential susceptibility factors worthy of investigation.

#### Dual subject studies

The past decade witnessed a tendency for coupled/comparative studies, in which the results obtained from avian and human subjects were jointly analyzed. Most of these studies tackled different behavioral features of diagnostic or therapeutic significance of ASD (Koshiba et al., 2016; Kelley et al., 2017; Galizio et al., 2020; Shvarts et al., 2020). In addition, molecular neuroanatomical studies, carried out on multiple species, including man, have also been reported, e.g., for the comparative localization of FMRP (see above) in the auditory system (Wang et al., 2014). Dual subject reports further highlight the translational importance of investigation into mechanisms across species, in which birds have a fair and growing share.

Notion from comprehensive animal experiments will likely be extrapolated to normal and impaired regulation of social behaviors in humans. By model building of causal and therapeutic significance, avian experiments continue to contribute toward the elucidation of anatomically traceable neural systems and developmental factors underlying human autism spectrum disorder.
